# Duration of Reproductive Period and Risk of Transitioning to Mild Cognitive Impairment and Dementia

**DOI:** 10.1093/geroni/igab046.3483

**Published:** 2021-12-17

**Authors:** Tomiko Yoneda, Jamie Knight, Nathan Lewis, Graciela Muniz-Terrera, David Bennett, Andrea Piccinin

**Affiliations:** 1 University of Victoria, Victoria, British Columbia, Canada; 2 University of Edinburgh, Edinburgh, Scotland, United Kingdom; 3 Rush University, Chicago, Illinois, United States

## Abstract

Decreasing estrogen levels have been hypothesized to be associated with increased risk of dementia, yet the current literature reveals conflicting results. This study aimed to determine whether a longer reproductive period, as an indicator of longer exposure to endogenous estrogens, is associated with risk of transitioning to MCI and dementia. Women 65 and over (N=1507) from the Rush Memory and Aging Project met eligibility for the current analysis. The average length of reproductive period (menopause age minus menarche age) was 35 years (range=16-68 years), and 64% had natural menopause. Multistate survival modeling (MSM) was used to estimate the influence of reproductive period on risk of transitioning through cognitive states including mild cognitive impairment (MCI) and clinically diagnosed dementia, as well as death. Multinomial regression models estimated total and cognitively unimpaired life expectancies based on the transition probabilities estimated by the MSM. Results suggest that women with more reproductive years were less likely to transition from no cognitive impairment (NCI) to MCI, and were more likely to return to NCI from MCI. Analyses also suggest two additional years free of cognitive impairment for women with 45 vs 25 years of reproduction, though reproduction period did not significantly impact overall life expectancy. This study suggests that the number of years of reproductive duration is not associated with the transition to dementia, but is possibly associated with delayed cognitive decline, reduced risk of MCI, increased likelihood of returning to NCI from MCI, and increased lifespan free of cognitive impairment.

